# Electrochemical immunosensor based on superwettable microdroplet array for detecting multiple Alzheimer’s disease biomarkers

**DOI:** 10.3389/fbioe.2022.1029428

**Published:** 2022-10-18

**Authors:** Zhen Huang, Mifang Li, Lingyan Zhang, Yibiao Liu

**Affiliations:** ^1^ Longgang District Central Hospital of Shenzhen, Shenzhen, China; ^2^ Office of Shenzhen Clinical College, Guangzhou University of Chinese Medicine, Longggang District Central Hospital, Shenzhen, China

**Keywords:** superwettable electrochemical biosensor, vertical graphene, gold nanoparticles, Alzheimer’s disease, portable biosensors

## Abstract

Alzheimer’s disease (AD) is a neurodegenerative disease caused by neurons damage in the brain, and it poses a serious threat to human life and health. No efficient treatment is available, but early diagnosis, discovery, and intervention are still crucial, effective strategies. In this study, an electrochemical sensing platform based on a superwettable microdroplet array was developed to detect multiple AD biomarkers containing Aβ40, Aβ42, T-tau, and P-tau181 of blood. The platform integrated a superwettable substrate based on nanoAu-modified vertical graphene (VG@Au) into a working electrode, which was mainly used for droplet sample anchoring and electrochemical signal generation. In addition, an electrochemical micro-workstation was used for signals conditioning. This superwettable electrochemical sensing platform showed high sensitivity and a low detection limit due to its excellent characteristics such as large specific surface, remarkable electrical conductivity, and good biocompatibility. The detection limit for Aβ40, Aβ42, T-tau, and P-tau181 were 0.064, 0.012, 0.039, and 0.041 pg/ml, respectively. This study provides a promising method for the early diagnosis of AD.

## Introduction

Alzheimer’s disease (AD) is a long-term neurodegenerative disease, that places a heavy burden on individuals, families, and communities. (Scheltens et al., 2021). Up to now, no effective cure for AD is available. Early diagnosis and early intervention are still very effective and important measures. (Li et al., 2021; Scheltens et al., 2021; Alzheimer’s Association 2022). At present, the gold standards for AD diagnosis are positron emission tomography (PET) and the level of biomarkers, including β-amyloid (Aβ) peptide and tau protein, in cerebrospinal fluid (CSF). (Marcus et al., 2014; Scheltens et al., 2021). However, AD diagnosis based on PET or CSF biomarkers is inapplicable to AD screening of the general population due to its high cost and invasive nature. Early diagnosis of AD based on blood biomarkers has elicited increased attention in recent years, and many studies have shown that AD can be diagnosed by measuring quantitative blood biomarkers, such as Aβ40, Aβ42, T-tau, and P-tau181. (Nakamura et al., 2018; Startin et al., 2019; Kim et al., 2020a; Janelidze et al., 2020; Thijssen et al., 2021; Mielke et al., 2022; Moscoso et al., 2022; Rubin 2022). However, the physiological concentration of AD blood markers, such as Aβ40, Aβ42, T-tau, and P-tau181, is only at the picograms level per milliliter. This concentration exceeds the detection limit of the conventional enzyme-linked immunosorbent assay (ELISA). Therefore, developing low-cost, non-invasive, and highly-sensitive detection methods for AD blood biomarkers is essential. (Nakamura et al., 2018; Brazaca et al., 2020). Thus far, many analytical methods have been developed to measure AD biomarkers in the blood, and these include electrochemistry (Liu et al., 2015; Liu et al. 2022a; Liu et al. 2022b; Zhang et al., 2022), fluorescence (Li et al., 2018; Zhang and Tan 2022), colorimetry (Duan et al., 2020), surface enhanced Raman spectroscopy (SERS) (Ma et al., 2021; Yang et al., 2022), and field-effect transistors (Sun Sang et al., 2021). Among these methods, electrochemical biosensors have great potential for disease diagnosis due to their easy miniaturization, high sensitivity, and low cost.

Superwettable microchips integrate two extremes of superhydrophobicity and superhydrophilicity into a 2D micropatterns (Xu et al., 2019), and are widely applied in biological medicine (Popova et al., 2015; Leite et al., 2017) and biochemical analysis (Xu et al., 2015; Xu et al. 2017; Xu et al. 2018) due to their outstanding ability for patterning microdroplets. In biosensing, superwettable microchips have remarkable advantages, including good microdroplet anchoring ability, low sample usage, high throughput, and enrichment ability. In addition, superwettable microchips can be combined with various signal output approaches, such as electrochemistry (Zhang et al., 2017; Song et al., 2019; Zhu et al., 2022), fluorescence (Chen et al., 2018), colorimetry (Hou et al., 2015; Xu et al., 2017; Zhang et al., 2021), and SERS (Song et al., 2018).

**FIGURE 1:**
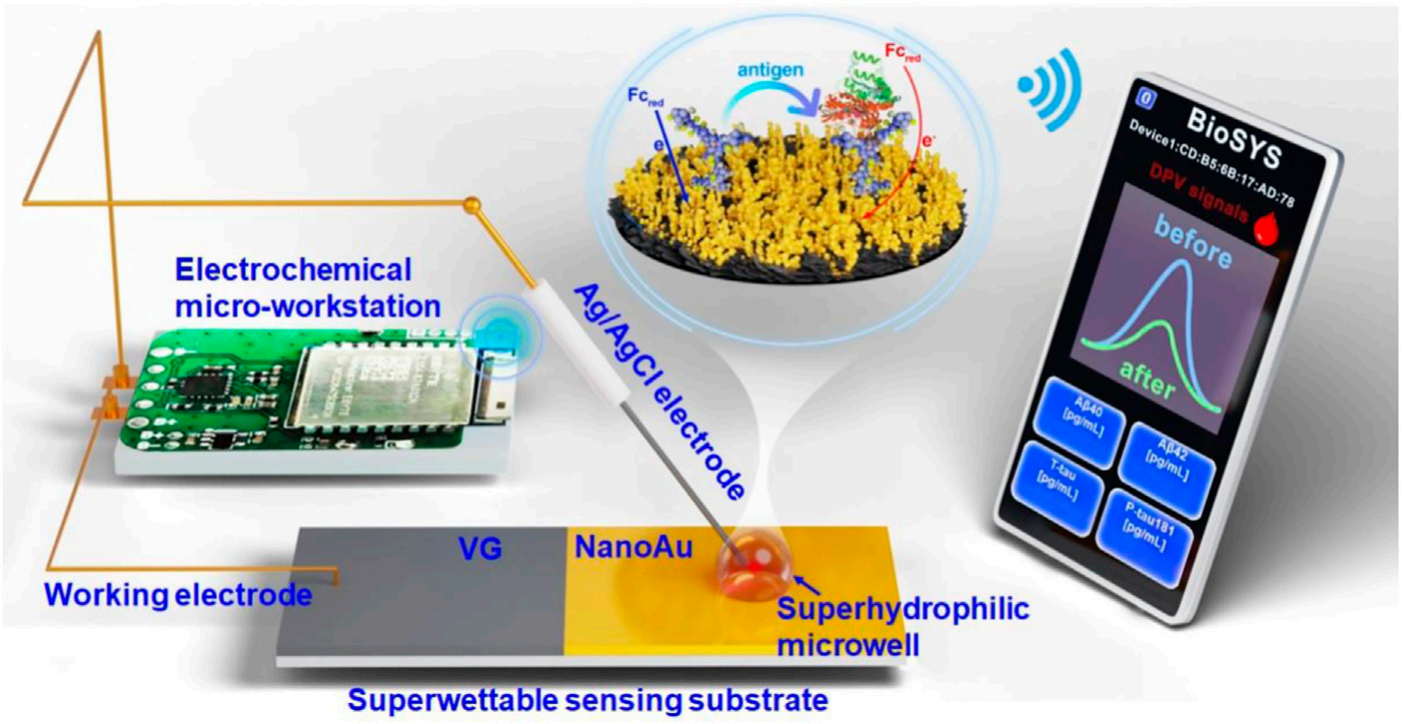
Schematic of the superwettable electrochemical sensing platform for AD biomarkers.

In this study, we integrated a superwettable substrate into an electrochemical biosensor, and developed a portable superwettable electrochemical sensing platform for the detection of multiple AD blood biomarkers. As shown in Figure 1, this portable sensing platform is composed of a superwettable sensing substrate, an electrochemical micro-workstation, and a smartphone. The superwettable substrate contains superhydrophilic microwell regions and superhydrophobic regions. The antibody of the target protein was fixed to the superhydrophilic microwell region by Au-S. Then, BSA was used to block the nonspecific binding sites. The peak current of differential pulse voltammetry (DPV) further decreased after binding with the target antigen. The peak current of DPV was recorded, and the target protein concentration was calculated according to the peak current change value. The electrochemical micro-workstation and smartphone were used to regulate and control electrochemical signals. The superwettable electrochemical sensing platform used a two-electrode system. Ag/AgCl electrode served as the reference and counter electrodes. NanoAu-modified vertical graphene (VG) was used as the working electrode. The design of the microdroplet system significantly reduced the use of the sample. A real picture of this portable sensing platform was shown in Supplementary Figure S1. The superwettable microchip also showed an enrichment ability in some ways, and decreased the detection limit (LOD). As a result, the superwettable electrochemical sensing platform exhibited a wide linear range and low LOD. This work offers great potential for the early diagnosis of AD.

## Experimental section

### Chemicals and materials

Aβ peptides (including Aβ40 and Aβ42), human serum albumin (HSA), glucose (GLU), potassium chloride (KCl), potassium ferricyanide/ferrocyanide (K_3_ [Fe(CN)_6_]/K_4_ [Fe(CN)_6_]), ferrocene, and phosphate-buffered solution (PBS, pH = 7.4, 10 mM) were purchased from Sigma-Aldrich (Shanghai, China). T-tau, P-tau181 protein, bovine serum albumin (BSA) and Aβ antibody were purchased from Abcam Ltd (Hong Kong, China). The antibodies of T-tau and P-tau181 were obtained from Thermo Fisher Scientific Co., Ltd. (Beijing, China). The commercial goat serum (Gibco) was purchased from Thermo Fisher Scientific Co., Ltd. (Beijing, China). All chemical reagents were of analytical grade. All solutions were prepared with ultrapure water (Milli-Q, 18.2 MΩ).

### Characterization and measurement

The morphology and elemental mapping of VG and VG modified with nanoAu were characterized through field-emission scanning electron microscopy (SEM, ThermoFisher, FEI Apreo S, Waltham, MA, United States). Water contact angles (CA) were measured at room temperature with a DSA100S system (KRUSS, Germany). All electrochemical measurements were performed on a customized electrochemical micro-workstation (Refresh AI Biosensor Co., Ltd., Shenzhen, China) at room temperature.

#### Construction of superwettable electrochemical substrate

First, VG on a ceramic surface was prepared through chemical vapor deposition (CVD). Second, nanoAu was modified on the VG surface through the electrodeposition of 10 mM HAuCl_4_. The deposition voltage was -1.8 V, and deposition time was 300 s. Third, the VG substrate modified with nanoAu was immersed in a n-decanethiol solution for 24 h at room temperature, and n-decanethiol was fixed on the surface of nanoAu. Lastly, the nanoAu modified with n-decanethiol substrate was treated with 120 s O_2_ plasma to obtain a superwetting electrochemical substrate containing superhydrophobic and superhydrophilic regions.

#### Preparation of electrochemical sensing platform based on superwettable substrate

After preparing the superwettable substrate, a superwettable electrochemical biosensor was constructed. First, 5 μL of the antibody of target protein (Aβ40, Aβ42, T-tau, and P-tau181) was dripped onto the superhydrophilic microwell region and incubated at 37°C for 1 h. Second, 5 μL of bovine serum albumin (BSA, 1%) was dropped onto the microwell, which was incubated for 1 h, and used to block the nonspecific binding sites. Third, 5 μL of different concentrations of the target protein was added to the superhydrophilic microwell surface, and incubated for 1 h at 37°C. After each step, the microwell surface was washed three times with PBS (0.01 M, pH = 7.4). Lastly, by combining the superwettable electrochemical substrate with the electrochemical micro-workstation, a superwettable electrochemical sensing platform was successfully constructed.

The target protein was measured *via* DPV by using a portable electrochemical micro-workstation. The working potential of DPV was in the range of 0–0.4 V. After incubation with different concentrations of the target protein, the corresponding peak current change value (ΔI) was recorded, and used to calculate the concentration of the target protein. The selectivity of this superwettable electrochemical sensing platform was investigated in PBS buffer containing HSA, GLU, Aβ40, Aβ42, Tau441, and P-tau181.

#### The application of the superwettable electrochemical sensing platform in goat serum.

The performance of the portable superwettable electrochemical sensing platform in goat serum was characterized through DPV. In brief, the antibody of the target protein (5 μL) was immobilized on the superhydrophilic microwell surface. Then 5 μL of commercial serum samples (1 μL goat serum diluted with 4 μL PBS buffer) containing different concentrations of the target protein (1, 10, and 100 pg/ml) was added to each superhydrophilic microwell surface and incubated at 37 °C for 1 h. The peak current change value of the DPV signal was monitored in this process.

#### Detection of clinical serum samples

5 μL clinical human sample (1 μL sample diluted with 4 μL PBS buffer) was added to the superhydrophilic microwell surface and incubated for 1 h at 37 C. The peak current value of DPV signals was recorded, and the target protein concentration was calculated according to the peak current changing value.

## Results and discussion

### Preparation and characterization of superwettable substrate

**FIGURE 2:**
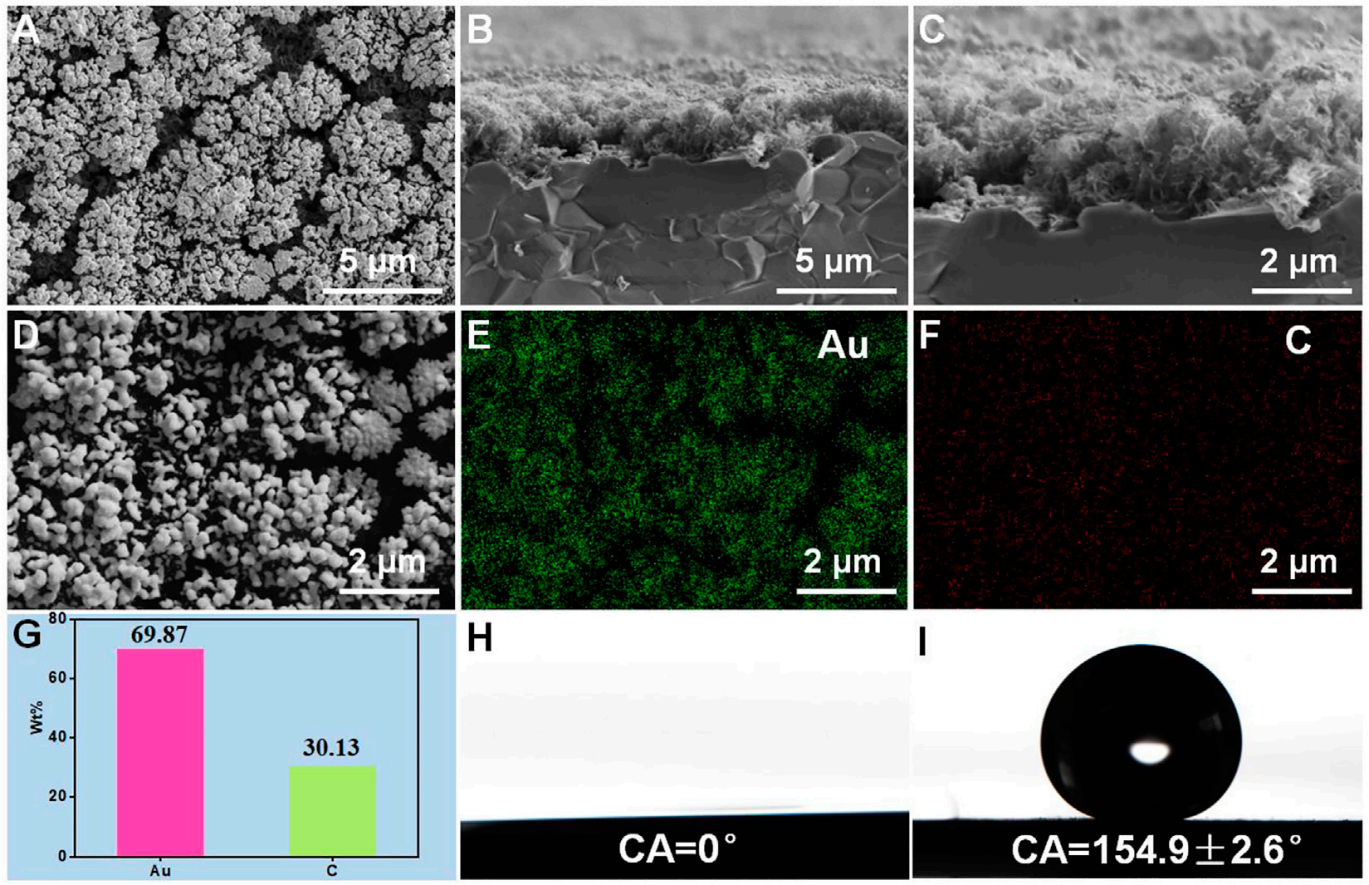
Surface **(A)** and cross-section view **(B,C)** SEM images of vertical graphene modified with nanoAu. Surface element distribution characterization **(D–G)** of vertical graphene modified with nanoAu. Water contact angles of the superhydrophilic region **(H)** and superhydrophobic region **(I)**.

The fabrication of the superwettable substrate is shown in Supplementary Figure S2. NanoAu was modified on the surface by electrodeposition, which could increase the electron transfer rates and improve the sensitivity of the senor. N-decanethiol was immobilized on the nanoAu surface, and the water contact angle was 154.9 ± 2.6°, indicating a superhydrophobic surface. After treatment by O_2_ plasma for 120 s, the water contact angle became 0°, indicating that the region without photomask became superhydrophilic. (Figures 2H,I). A superwettable substrate that included superhydrophobic and superhydrophilic regions was successfully prepared.

The surface morphology of the superwettable substrate was evaluated *via* SEM, and the results are shown in Figure 2A. Many gold nanoparticles were observed on the surface of the layered vertical graphene structures. The cross-section view SEM images of vertical graphene was shown in Supplementary Figure S3. The cross-section view morphology of vertical graphene@Au is shown in Figures 2B,C. The gold nanoparticles were deposited mainly on the VG surface. In addition, the content of Au was measured through energy dispersive X-ray (EDX), and the result showed that the weight percent of Au element was 69.87%, indicating that most of the surface areas of VG were covered with Au nanoparticles (Figures 2D– G).

### Construction and analytical performance of superwettable electrochemical sensing platform

**FIGURE 3:**
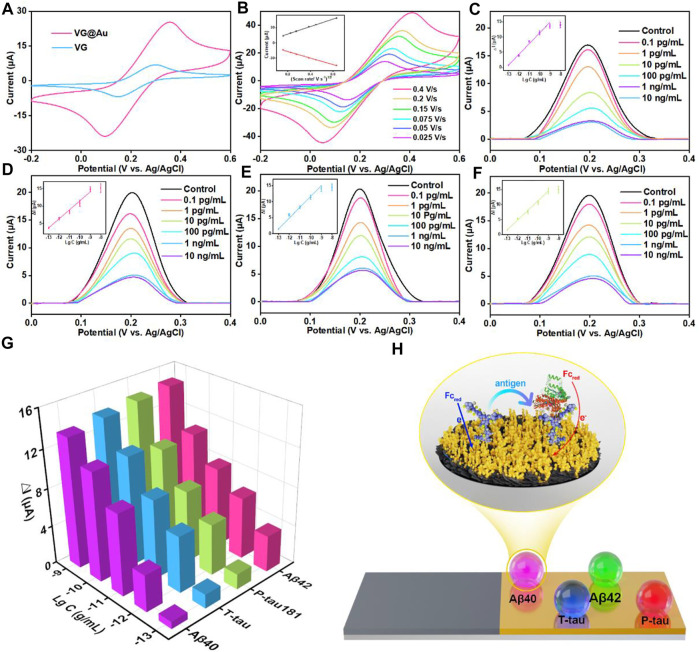
Analytical performance of the superwettable electrochemical sensing platform based on VG@Au for the detection of AD biomarkers **(A)** CVs of VG and VG@Au electrode in 5 mM [Fe(CN)_6_]^3−^/[Fe(CN)_6_]^4−^ solution containing 0.1 M KCl at 0.1 V/s. **(B)** CVs of the superwettable microwell electrode based on VG@Au at different scan rates. The inset is the relationship between the peak current and the square root of scan rate. DPV signals of different concentrations of Aβ40 **(C)**, Aβ42 **(D)**, T-tau **(E)**, and P-tau181 **(F)** in 1 mM ferrocene solution containing 0.1 M KCl at 0.1 V/s. **(G)** Change value (ΔI) of DPV response signals towards AD biomarkers. **(H)** Schematic of the electrochemical immunosensor based on VG@Au.

The electroactive areas of VG and VG@Au were compared by cyclic voltammetrys (CVs), and the results showed that the electroactive area of the VG@Au electrode was remarkably larger than that of the VG electrode. (Figure 3A). In addition, the electrode surface dynamics process was evaluated by CV at different scan rates. As shown in Figure 3B, the peak current had a linear relation with the square root of the scan rate, indicating a diffusion-limited process. After the superwettable substrate was completed, the antibody of the target protein (Aβ40, Aβ42, T-tau, and P-tau181) was immobilized on the superhydrophilic microwell region by Au-S. After modifying the corresponding antibody, the peak current of the DPV signal decreased, which indicated that the target protein antibody was successfully fixed on the superwettable microwell region surface (Supplementary Figure. S4, lines I and II). Then, BSA was used to block the nonspecific adsorption sites, and the peak current of the DPV signals further decreased (Supplementary Figure S4, line III). Afterward, the different concentrations of the target protein were added to the superhydrophilic microwell region surface. During this period, the DPV signal was recorded, and the concentration of the target protein was calculated based on the variation of the peak current.

The concentration of the target protein antibody was optimized before the final test. As shown in Supplementary Figure S5, the optimized concentration of the target protein antibody was 10 μg/ml. Under the optimized condition, the antibody of four proteins was immobilized in the superhydrophilic microwell regions, and the concentration of the target proteins was measured by the variation in the resistance of the superhydrophilic microwell region surface, as shown in Figure 3H. The corresponding antibody specifically recognized the target protein (Aβ40, Aβ42, T-tau, and P-tau181), resulting in the increase in surface resistance, which caused signal reduction. The peak current of the DPV signals was recorded by the electrochemical micro-workstation platform, and the concentration of the target protein was calculated by the variation in peak current.

As shown in Figures 3C–G, for the target protein (Aβ40, Aβ42, T-tau, and P-tau181), the peak current of DPV signals decreased, and the variation in peak current (ΔI) increased as the concentration of the target protein increased. ΔI had a good linear relationship with the logarithm of target protein concentration from 0.1 pg/ml to 1,000 pg/ml. The detection limit of this superwettable electrochemical sensing platform for Aβ40 was about 0.064 pg/ml (S/N = 3). The LOD was calculated as three times the standard deviation of the blank. (Chiavaioli et al., 2017; Esposito et al., 2021). Similarly, the LOD for Aβ42, T-tau, and P-tau181 was 0.012, 0.039, and 0.041 pg/ml, respectively.

In blood, the physiological concentration of Aβ40, Aβ42, T-tau, and P-tau181 was about several to hundreds of picograms per milliliter. This result shows that our developed superwettable electrochemical sensing platform satisfies the needs of detecting AD biomarkers in blood. The superwettable electrochemical sensing platform based on the VG@Au array exhibited a low LOD and a wide linear range. A comparison of this method and methods in previous reports is shown in Table 1. Our developed superwettable electrochemical sensing platform exhibited excellent analytical performance. The concentration of AD biomarkers including Aβ40, Aβ42, T-tau, and P-tau181, was at picograms per milliliter of blood. The LOD of this superwettable electrochemical sensing platform was lower than 0.1 pg/ml, which meets the requirements for the detection of AD biomarkers.

### Selectivity and stability

**TABLE 1:** Comparison between the superwettable electrochemical sensing platform and other sensors for the detection of AD biomarkers.

Method	Target	Detection limit	References
Electrochemistry	Tau, p-tau181, Aβ42, Aβ40	2.45, 2.72, 2.13, 2.20 fM	Kim et al. (2020b)
Electrochemistry	ApoE4, Tau, Aβ	5.91 × 10^−11^;7.1 × 10^−11^; 8.6 × 10^−12^ mg/ml	Song et al. (2020)
Electrochemistry	T-tau, p-tau181, Aβ40, Aβ42	0.125, 0.089, 0.142, 0.176 pg/ml	Liu et al. (2022a)
Fluorescence	Aβ42, tau441, p-tau181	340.07, 669.44, 493.79 pg/ml	Chan et al. (2017)
LSPR	Aβ40, Aβ42, T-tau,	34.9, 26, 23.6 fM	Kim et al. (2018)
SERS	Tau, Aβ42 oligomers	4.2 × 10^–4^ pM, 3.7 × 10^–2^ nM	Zhang et al. (2019)
FET sensor	tau	10 fg/ml	Sun Sang et al. (2021)
Electrochemistry	T-tau, p-tau181, Aβ40, Aβ42	0.039, 0.041, 0.064, 0.012 pg/ml	This work

Selectivity and stability.

**FIGURE 4:**
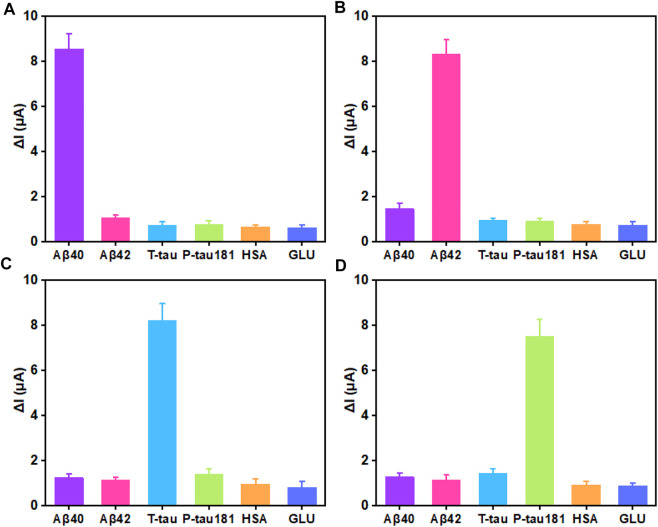
Selectivity of the superwettable electrochemical sensing platform **(A)** Variation of the peak current of 10 pg/ml Aβ40 and 1 ng/ml Aβ42, T-tau, P-tau181, GLU, and HSA. **(B)** Variation of the peak current of 10 pg/ml Aβ42 and 1 ng/ml Aβ40, T-tau, P-tau181, GLU, and HSA **(C)** Variation of the peak current of 10 pg/mL T-tau and 1 ng/ml Aβ40, Aβ42, P-tau181, GLU, and HSA. **(D)** Variation of the peak current of 10 pg/mL P-tau181 and 1 ng/ml Aβ40, Aβ42, T-tau, GLU, and HSA.

In biological application, selectivity and stability are important factors for biosensors. The selectivity and stability of this superwettable electrochemical sensing platform were investigated. As shown in Figure 4A, when 10 pg/ml of Aβ40 was added, an obvious signal response of 8.56 μA was obtained. On the surface of the superhydrohilic microwell sensing region, the concentration of other proteins including Aβ42, T-tau, P-tau181, GLU, and HSA was 100-fold higher than that of Aβ40, and the △I for Aβ42, T-tau, P-tau181, GLU, and HSA was 1.06, 0.73, 0.75, 0.66, and 0.62 μA, respectively, which accounted for 12.4%, 8.5%, 8.7%, 7.7%, and 7.2% of the △I for Aβ40, respectively. For Aβ42 sensing region, the signal response was about 8.33 μA. The △I for Aβ40, T-tau, P-tau181, GLU, and HSA was 1.46, 0.96, 0.89, 0.78, and 0.73 μA, respectively, which accounted for 17.5%, 11.5%, 10.6%, 9.3% and 8.7% of the △I for Aβ42, respectively (Figure 4B). Likewise, for T-tau or P-tau181, the corresponding superhydrohilic microwell sensing region also displayed excellent selectivity (Figures 4C,D). These results indicate that the selectivity of this superwettable electrochemical sensing platform for Aβ40, Aβ42, T-tau, and P-tau181 was outstanding. In addition, the stability of this superwettable electrochemical sensing platform was evaluated by detecting six times of 10 pg/ml of the target protein (Aβ40, Aβ42, T-tau, and P-tau181) As shown in Supplementary Figure S6, the sensor was stored in dry conditions at 4°C for 2 weeks. The ΔI value still remained above 90% of its initial value after 14 days, demonstrating the acceptable stability of this superwettable electrochemical sensing platform based on VG@Au. The results prove that our developed electrochemical sensing platform based on a superwettable microarray has good stability and specificity.

### Application of this superwettable electrochemical sensing platform in serum sample

To further evaluate the clinical application of the superwettable electrochemical sensing platform, goat serum samples that included Aβ40, Aβ42, T-tau, and P-tau181 were detected by using the designed superwettable platform. The diluted serum samples were spiked with different concentrations of the target protein (1, 10, and 100 pg/ml) including Aβ40, Aβ42, T-tau, and P-tau181. The result was shown in Supplementary Table S1. No significant difference was observed between the detected and added values. The recovery rate ranged from 91% to 109.1%. In addition, we conduct two clinical samples and compared the results with the results from typical ELISA. As shown in Supplementary Table S2, Aβ40 and Aβ42 can be detected by this superwettable sensor and typical ELISA, there was no significant difference between the result of our sensor and that of ELISA. This result demonstrated that our developed superwettable electrochemical sensing platform based on VG@Au could be used for detecting the clinical samples. What’s more, T-tau and P-tau181 were detected by this sensor, but they were not detected by typical ELISA, indicating that this superwettable electrochemical sensor has lower LOD. To sum up, the superwettable electrochemical sensing platform based on VG@Au has excellent sensitivity and reliability for the detection of AD biomarkers in clinical serum sample analysis, and could be capable of clinical diagnosis.

## Conclusion

In summary, a portable superwettable electrochemical sensing platform based on the VG@Au substrate was designed and constructed to detect multiple AD biomarkers in serum. The superwettable VG@Au substrate included superhydrophobic and superhydrophilic regions on the VG@Au surface, which could be used for fixing a microdroplet sample, and used as a working electrode to generate electrochemical signals. In addition, an electrochemical micro-workstation was introduced to this superwettable electrochemical sensing platform to adjust the signal. The superwettable electrochemical sensing platform based on the superwettable VG@Au substrate showed excellent analytical performance with a low detection limit and high sensitivity due to the good properties of VG@Au, including large specific surface, outstanding electrical conductivity, and good biocompatibility. As a result, the detection limit for Aβ40, Aβ42, T-tau, and P-tau181 were 0.064, 0.012, 0.039, and 0.041 pg/ml, respectively. In blood, the AD biomarker concentration was at the ∼pg/mL level. Our designed superwettable sensing platform satisfies the need for detection in blood. This work offers a new method of detecting AD biomarkers in serum. The method exhibites great potential for early diagnosis of AD.

## Data Availability

The original contributions presented in the study are included in the article/Supplementary Material, further inquiries can be directed to the corresponding authors.
