# Supplementary material for: Electrochemical immunosensor based on superwettable microdroplet array for detecting multiple Alzheimer’s disease biomarkers
Source: Front Bioeng Biotechnol. 2022 Oct 18;10:1029428. doi: 10.3389/fbioe.2022.1029428 (PMC9622762; doi:10.3389/fbioe.2022.1029428)
Supplement: Supplementary file 1 [file DataSheet1.docx]

**Supplementary Information**

**Electrochemical immunosensor based on superwettable microdroplet array for detecting multiple Alzheimer’s disease biomarkers**

Zhen Huang^a, b #^, Mifang Li^a #^, Lingyan Zhang^a,^ *, Yibiao Liu^a, b^*

^a^ Longgang District Central Hospital of Shenzhen, Shenzhen, China

^b^ Office of Shenzhen Clinical College, Guangzhou University of Chinese Medicine, Longggang District Central Hospital, Shenzhen, China

# contributed equally to this work

E-mail: [18819818005@163.com](mailto:18819818005@163.com); [liuyibiao12345@126.com](mailto:liuyibiao12345@126.com);


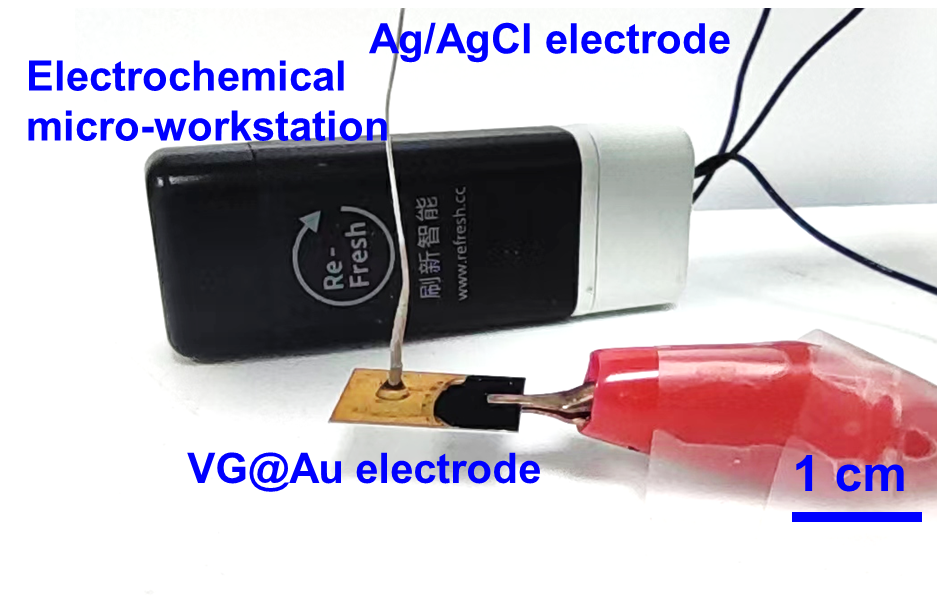


**Figure S1**. A real picture of the superwettable electrochemical sensing platform.


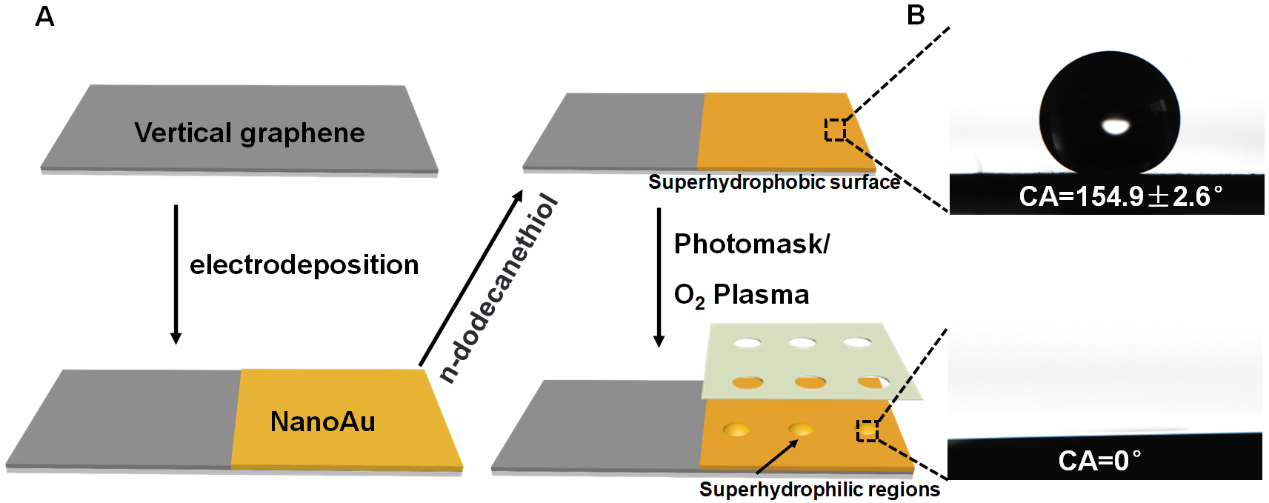


**Figure S2.** (A) Fabrication of superwettable microchips based on vertical graphene modified with nanoAu. (B) Water contact angle of the superhydrophobic and superhydrophilic surface.


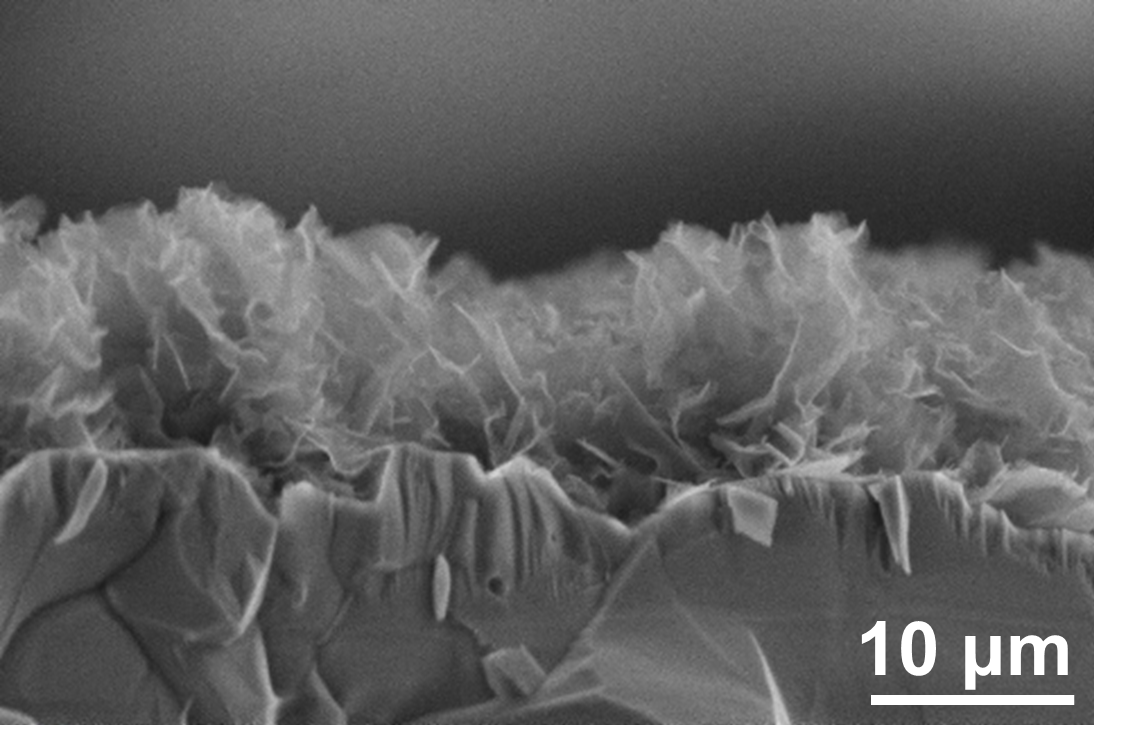


**Figure S3**. The cross-section view SEM images of vertical graphene.


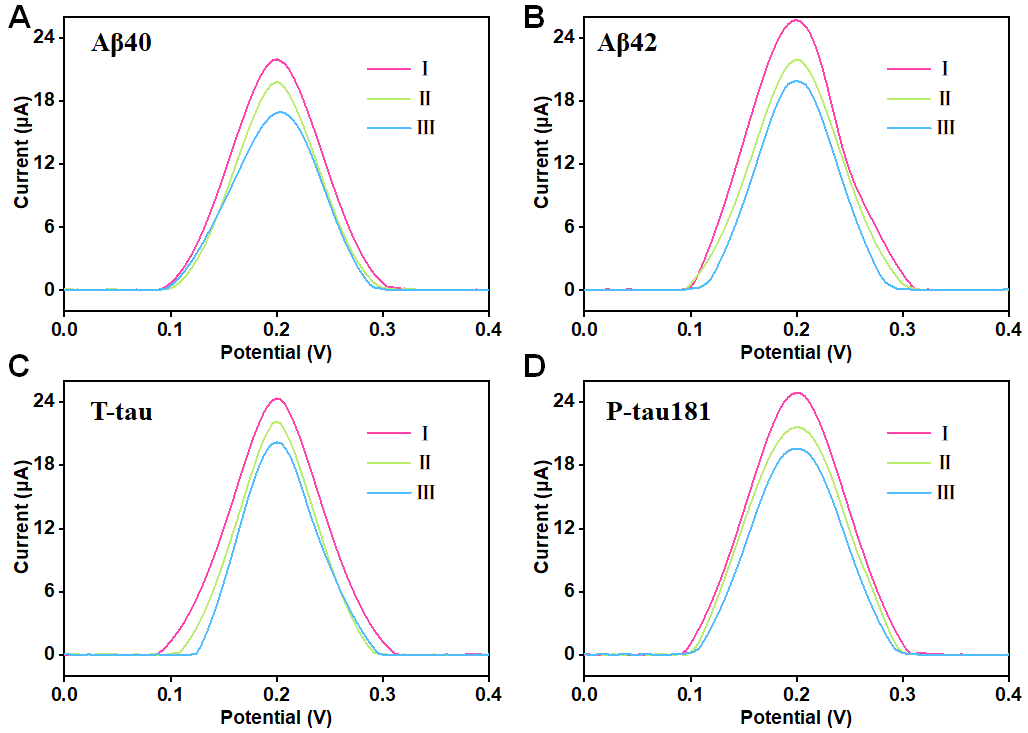


**Figure. S4** (A)DPV signals of the VG@Au electrode (curve Ⅰ) modified with Aβ40 antibody (curve Ⅱ), Aβ40 antibody + BSA (curve Ⅲ). (B) DPV signals of the VG@Au electrode (curve Ⅰ) modified with antibody of Aβ42 antibody (curve Ⅱ), Aβ42 antibody + BSA (curve Ⅲ). (C) DPV signals of the VG@Au electrode (curve Ⅰ) modified with T-tau antibody (curve Ⅱ), T-tau antibody + BSA (curve Ⅲ). (D)DPV signals of the VG@Au electrode (curve Ⅰ) modified with P-tau181 antibody (curve Ⅱ), P-tau181 antibody + BSA (curve Ⅲ).The DPV are carried out in ferrocenyl methanol at scan rates of 0.1 V‧s^-1^.


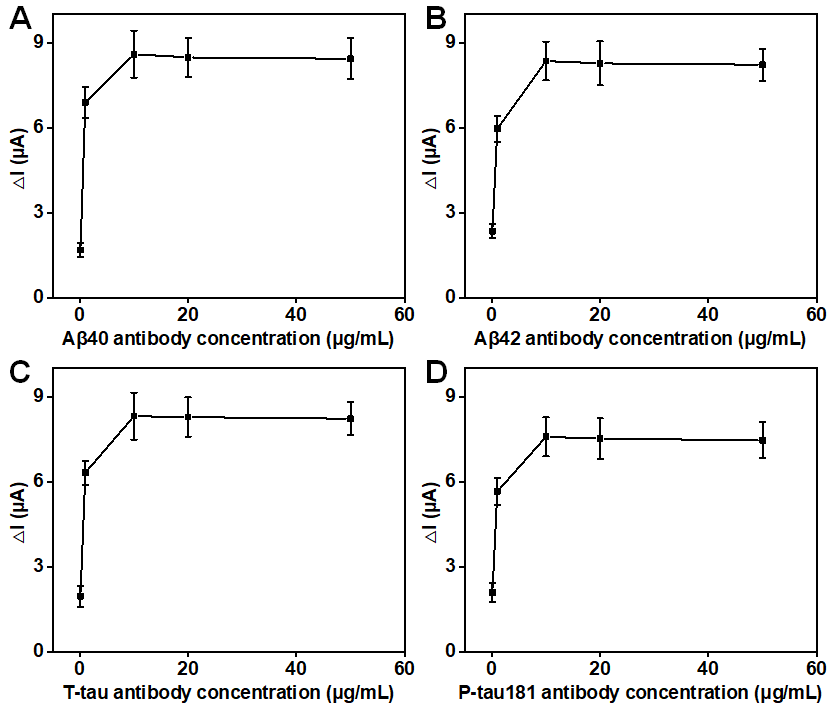


**Figure. S5** Optimization of target protein antibody concentration.


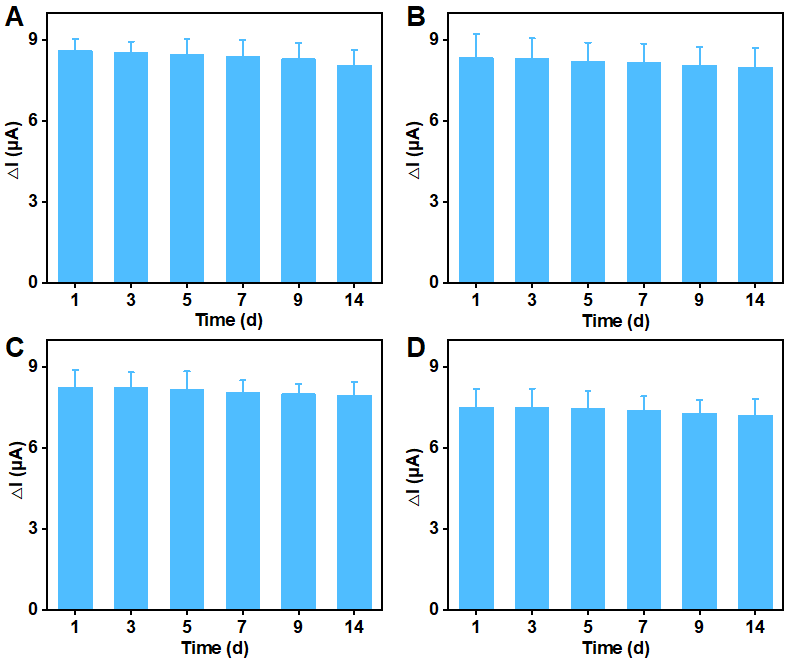


**Figure S6.** Stability of the sperwettable electrochemical sensing platform.

**Table S1.** Recovery study for detecting AD biomarkers in commercial goat serum.

| biomarkers | C_added_ (pg/mL) | C_founded_ (pg/mL) | Recovery (%) |
| --- | --- | --- | --- |
|  | 1 | 0.91 | 91.0 |
| Aβ40 | 10 | 10.96 | 109.6 |
|  | 100 | 105.6 | 105.6 |
|  | 1 | 1.09 | 109.0 |
| Aβ42 | 10 | 9.31 | 93.1 |
|  | 100 | 92.2 | 92.2 |
|  | 1 | 1.06 | 106.0 |
| T-tau | 10 | 10.33 | 103.3 |
|  | 100 | 109.8 | 109.8 |
|  | 1 | 0.95 | 95.0 |
| P-tau181 | 10 | 10.75 | 107.5 |
|  | 100 | 91.3 | 91.3 |

Table S2. Comparison between this superwettable electrochemical sensor and typical ELISA for AD biomarkers in clinical serum sample.

| Sample | Biomarkers | Average measured concentration (pg/mL) | |
| --- | --- | --- | --- |
|  |  | This superwettable  electrochemical sensor | ELISA |
| 1 | Aβ40 | 274.20±9.56 | 268.50±8.48 |
|  | Aβ42 | 11.77±0.98 | 11.98±0.93 |
|  | T-tau | 2.21±0.12 | / |
|  | P-tau181 | 1.62±0.16 | / |
| 2 | Aβ40 | 395.39±14.87 | 379.37±10.54 |
|  | Aβ42 | 14.38±1.23 | 15.08±0.92 |
|  | T-tau | 4.11±0.16 | / |
|  | P-tau181 | 2.98±0.13 | / |
